# A Component-Based Approach for Securing Indoor Home Care Applications

**DOI:** 10.3390/s18010046

**Published:** 2017-12-26

**Authors:** Aitor Agirre, Aintzane Armentia, Elisabet Estévez, Marga Marcos

**Affiliations:** 1 ICT Department, IK4-Ikerlan, 20500 Arrasate-Mondragón, Spain; 2 Automatic Control & Systems Engineering Department, ETSI Bilbao, University of the Basque Country (UPV/EHU), 48013 Bilbao, Spain; marga.marcos@ehu.eus; 3 Electronic and Automation Engineering Department, University of Jaen (UJA), 23071 Jaén, Spain; eestevez@ujaen.es

**Keywords:** eHealthcare, security, safety, reliability, service component architecture, data distribution service, domain modeling

## Abstract

eHealth systems have adopted recent advances on sensing technologies together with advances in information and communication technologies (ICT) in order to provide people-centered services that improve the quality of life of an increasingly elderly population. As these eHealth services are founded on the acquisition and processing of sensitive data (e.g., personal details, diagnosis, treatments and medical history), any security threat would damage the public’s confidence in them. This paper proposes a solution for the design and runtime management of indoor eHealth applications with security requirements. The proposal allows applications definition customized to patient particularities, including the early detection of health deterioration and suitable reaction (events) as well as security needs. At runtime, security support is twofold. A secured component-based platform supervises applications execution and provides events management, whilst the security of the communications among application components is also guaranteed. Additionally, the proposed event management scheme adopts the fog computing paradigm to enable local event related data storage and processing, thus saving communication bandwidth when communicating with the cloud. As a proof of concept, this proposal has been validated through the monitoring of the health status in diabetic patients at a nursing home.

## 1. Introduction

Information and communication technologies are gaining increasing importance in modern public health management. These technologies bring several benefits to patients, care teams, organization infrastructure (e.g., hospitals, clinics, home, etc.) and to the overall health environment, composed by regulators, insurers, health care purchasers, research funders, etc. [1,2,3]. On the one hand, the patients can benefit from faster, convenient, and more efficient medical processes, which result in improved health care. For example, real time and continuous monitoring of physiological parameters facilitate the early detection of health diseases and medical urgencies. Furthermore, this improved health tracking is achieved in a non-invasive and unattended manner, a fact that takes on special relevance when dealing with elderly patients. On the other hand, from the perspective of public health system management, a timely and targeted prevention supported by information and communication technologies results in general health care cost containment [4]. Other opportunities offered by e-Healthcare systems include early detection of pandemic [5,6]. The supporting technologies for these useful applications range from social networking [7] to big data [8] or search engines like Google Flu Trends [9].

To achieve such benefits, current trends in health care policy emphasize the active participation of patients in their own health care, in contrast to a more traditional view in which the patient was seen as a passive stakeholder. This patient driven approach implies access to a common *data bus* that is shared between the patient, the medical staff, the health care organization, and the political institutions that manage the overall health system. Obviously, such health care management approach implies several technical aspects that must be addressed. In concrete, two of them arise as particularly challenging: safety and security. *Safety* must be considered as mandatory when dealing with aspects such as computerized prescribing and medication [10] or remote monitoring of physiological data [11]. In this case, the integrity of the transmitted data must be protected against eventual data corruption. On the other hand, *privacy* and *security* are key aspects to be considered when dealing with personal health related sensitive data [12,13,14]. As far as the considered e-Health management system is intrinsically distributed, safety and security aspects must be covered from both data distribution and data persistence perspectives. This implies the design of reliable and secure communication channels, alongside with dependable data persistence mechanisms. 

Additional technical aspects that must be considered include, among others: (1) support for applications that are composed by mobile and dynamic elements. In this sense, as the system scales, more users (patients) can be registered in the system and these patients can be monitored by mobile devices such as wearable sensors or mobile phones that can dynamically appear or disappear in the healthcare management network. (2) Interoperability of systems and devices [15]. These devices are geographically distributed and deployed over heterogeneous networks and protocols. Examples of such systems and devices include wireless medical devices deployed in the home care network, wearable sensors, home gateways, hospital databases and so on. (3) To enable personalized medicine, customization of health care applications is a key issue that must be considered in order to fit specific user needs. 

This paper proposes a component-based solution to cover several needs of eHealthcare applications. It provides support for the whole lifecycle of such applications, including the design, deployment and execution phases, and plays special attention on their security and safety needs, which can be configured by the application integrator in the design phase. Specific mechanisms to support both secure communications and secure data persistence are proposed. Also, the framework provides a generic way to specify, in the design phase, the application specific process data that should be persisted in order to perform further analysis such as e.g., early pandemic detection. To achieve such features, the Distributed Applications Management Platform (DAMP) presented in [16] has been extended with new functionalities. On the one hand, the platform now provides a generic mechanism to track application data and ensure its persistence in a database. This way, the medical domain specialist can define, in the application configuration phase, the specific application data that must be persisted in order to perform further analysis. On the other hand, security is addressed from two aspects. First, a secure channel to communicate application components is provided. Second, security and availability of the persisted application data is ensured, through the appropriate data encryption and replication mechanisms. 

Another contribution of this work is a methodology for the design and development of eHealthcare applications, which extends the domain modeling approach presented in [17] with support for the specification of component security. This methodology decouples the medical domain specialist tasks and the technology expert tasks. As a result, the former can focus on the functional specification of the target applications whilst the latter specifies non-functional aspects such as the security level of the component interconnections or the database redundancy. The proposal also provides a generic way to specify, in the design phase, the application specific process data that should be persisted in order to perform further analysis such as early pandemic detection. Once the application configuration is finished, the methodology also provides guidelines and templates to implement application code. These fill the gap between the application specification (platform independent) and the component applications managed by DAMP platform, which does not depend on any application domain.

The remainder of the paper is as follows: Section 2 presents the requirements of the targeted eHealthcare applications. Section 3 details the runtime support that is proposed to fulfill such requirements whilst Section 4 covers the proposed design and development methodology, emphasizing security and safety related features. Section 5 presents an assessment of the runtime platform performance and security aspects, focusing on computing resource consumption. Section 6 provides a review of related work, and finally, Section 7 presents the conclusions and points out some ideas for future work.

## 2. eHealthcare Application Demands

eHealthcare applications are mainly focused on the continuous and remote monitoring of physiological variables that implies collecting and processing patient data as well as generating reliable data to share between the patient and health care experts aiming at identifying early health deterioration. To achieve this goal it is necessary to integrate health devices and the software that allows their remote control, capture and data processing through communications networks. For example, using the terminology stated by the Continua Design Guidelines [18], let us consider a system consisting on a personal health device (e.g., a pulse oximeter) that communicates its measurements via Bluetooth to a personal health gateway (e.g., a health & fitness app on a smart phone or a home domotic controller) which in turn preprocesses the data and forwards them to a health care information system (e.g., the hospital server), using a protocol such as HL7 [19] to transfer the medical data payload.

Such a remote home care system requires the development of application modules that may be diverse in terms of implementation languages and could interoperate over heterogeneous physical networks (e.g., Bluetooth, Ethernet, 4G) and communication protocols (e.g., Simple Object Access Protocol—Web Service (SOAP-WS), REpresentational State Transfer (REST)). Moreover, specific non-functional requirements must be considered to provide a dependable system that copes with sensitive personal data [20]. In this sense, aspects such as safety, security or availability must be considered.

There are also flexibility requirements that are particularly relevant in this kind of eHealth-centered applications [17]. In this sense, concepts as adaptability to context changes or customization of applications to specific user needs must be properly supported.

Remote monitoring is one of the key functionalities of the target applications (R1). This remote monitoring should be tailored to the particular needs of the patient taking into account that detection of health impairment and reaction to abnormal situations must also be customized to particular patients (R2).

From the overall eHealthcare system management point of view, support for remote control and configuration of health devices and applications (R3) is needed. This is a key feature that must be addressed to properly support an efficient system operation and maintenance, as it seems obvious, for example, that the elderly people should not care about the configuration and control of their health care devices, as these services should be delegated in physicians and system operators. 

Usually, healthcare applications must interoperate with already existing systems through different networks and protocols. Furthermore, the system may require application migration from one platform to another. Thus, reusability, interoperability and heterogeneity issues must be properly handled (R4).

In this sense, global dissemination of medical data through a global network infrastructure is getting a current challenge [21]. Certainly, there is a need of analytical tools on massive digital data that would result in improving health care at individual, group and international level (R5). As an example, this dissemination of medical data would enable following up the global evolution of chronic diseases such as diabetes or even the early detection and supervision of epidemic and pandemic at a world level.

The adequate dependability levels must be established in terms of the availability degree desired (R6), taking into account the affordable costs vs. the assumable service levels for a specific use case. For example, in the previous use case composed of a Bluetooth pulse oximeter plus a home domotic gateway that communicates with the cloud server of the hospital, it seems reasonable to replicate the hospital server as far as an eventual server crash could affect to hundreds of patients. Nevertheless, other system devices (e.g., some personal health devices) do not demand so high availability.

The sensitive nature of the managed patient data demands reliable data transmission (R7) as well as secure data transmission (R8). The former ensures message integrity over non-safe channels [11] and protects against non-intentional data corruption, whereas the latter ensures that medical data related messages are properly identified, authenticated, and protected against deliberated data modifications. 

Finally, critical data must be kept secure and safe, to protect against accidental or willful modifications (R9). This issue is closely related to requirement R5, as far as it provides a trustable source of data for subsequent medical data trend analysis.

Therefore, it can be concluded that the target applications exhibit a common set of characteristics that demand the fulfillment of several requirements during their design, development and operation. These requirements are summarized in Table 1.

## 3. Distributed Applications Management Platform (DAMP)

To support the typology of applications specified in the previous section, a component-based solution is proposed. This approach considers that applications are composed by several components interconnected across a network. To illustrate the advantages of a component-based solution let us consider a case study related to diabetic patients. Diabetes is a chronic disease that is expected to be the seventh cause of death in 2030 [22]. It occurs when the pancreas does not produce enough insulin or when the body is not able to use the insulin produced. As a result, the concentration of glucose in the blood increases (hyperglycemia), which can lead to serious damage such as blindness, kidney failure or lower-extremity amputation. Additionally, diabetic people may also be in risk of having a low level of sugar in blood (hypoglycemia) as a result of, for example, a wrong dose of insulin or too much physical exercise, and with the following symptoms: sweating, sickness, heart palpitations, blurred vision, tremors, etc. In severe cases it can lead to loss of consciousness [23,24,25]. In fact, there are works focused on relating hypoglycemia with the risk of suffering cardiovascular diseases [25,26,27].

**Figure 1:**
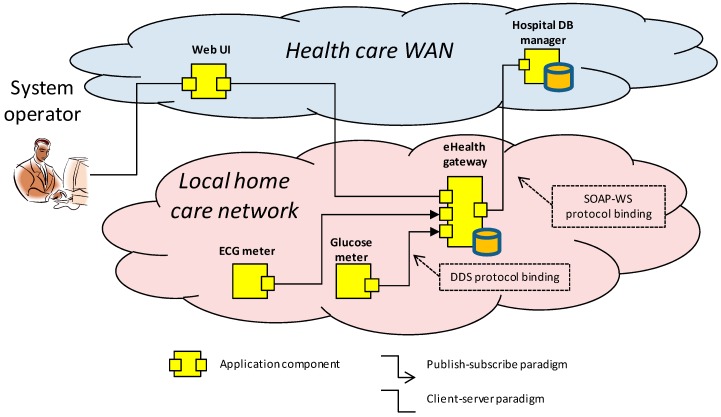
Health monitoring application.

In this context, the health monitoring application depicted in Figure 1 could be used to analyze the correlation between these two diseases. The yellow components make up a health monitoring application. The application periodically tracks the patient´s blood pressure and glucose level data. Initially, this data is stored locally in the eHealth gateway database. Then, periodically, it is compressed and uploaded to the hospital database. Once the monitored physiological parameters are stored in this database, further medical analysis can be performed in order to correlate hypoglycemic episodes with blood pressure data.

The periodic batch processing performed at the eHealth gateway optimizes the communication channel bandwidth. The gateway also centralizes the security related tasks: on the one hand, it securely stores local data. On the other hand, it performs the sign and encryption of the data that is remotely sent to the hospital database. 

Additionally, the gateway can be remotely parameterized to change the frequency of the batch processing or to perform security related tasks, such as cryptographic key renewal or encryption algorithm selection. This parameter tuning of the gateway is initiated by a remote operator through a web based user interface component, which provides centralized access to the home care networks of the different patients.

The example application represented in Figure 1 also highlights some common non-functional requirements discussed in the previous section. In particular, those related to heterogeneity and interoperability. In this sense, the example scenario uses the SOAP-WS protocol for the communication between the eHealth gateway and the Wide Area Network (WAN) components (i.e., hospital database manager and the web User Interface (UI)), whereas the selected communication protocol for the home Local Area Network (LAN) side is Data Distribution Service (DDS).

To support the execution of such kind of distributed applications, a runtime management platform called DAMP is proposed. DAMP manages application components based on Service Component Architecture (SCA) [28], which have been extended with additional control ports that enable its remote control. Section 3.1 explains briefly the SCA component model alongside with the DAMP platform architecture and services. Section 3.2 presents the API of the provided services, and explains in detail the stateful system recovery mechanisms that are key to support high availability in eHealthcare distributed applications. As a result, some of the requirements indicated in previous section are fulfilled. In particular, the **R1**, **R3**, **R4** and **R6** requirements described in Table 1.

To meet the rest of requirements, the DAMP platform has been leveraged in two ways. Section 3.3 describes the security mechanisms that guarantee the reliable and secure data transmission of sensitive medical data (**R7** and **R8**, in Table 1) between the application components. Section 3.4 details how the platform enables the possibility of specifying (in the design phase) the application data that needs to be stored for further Big Data Analysis (BDA) (**R5**), whilst guaranteeing its anonymity. Also, data encryption and database replication mechanisms are proposed to fulfill the **R9** requirement related to reliable and secure data persistence. It is worth noting that the configuration of all these aspects is performed in the integration phase, and therefore the functional aspects of the application are decoupled from those related to security, persistence, and privacy.

### 3.1. DAMP Architecture

The SCA standard defines a programming architecture oriented to the development of applications from building blocks (components) that encapsulate the business logic and offer it in the form of services. Thus, SCA offers a solution based on the Service Oriented Architecture (SOA) paradigm.

A SCA component consists of a language agnostic functional implementation (business logic) and a set of *properties*, *services*, and *references*. The SCA standard defines connections (*bindings*) for several implementation languages (C, C++, BPEL, Java and Spring), although depending on the specific SCA distribution, additional languages may be available [29]. 

**Figure 2:**
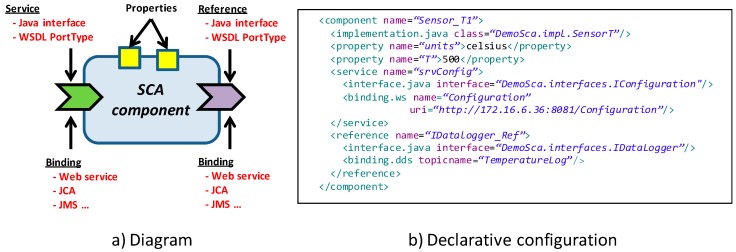
SCA components.

In the SCA nomenclature, a *service* refers to a functionality provided by the component, whereas a *reference* represents a functionality required by the component. As it is depicted in Figure 2a, services and references are depicted on the left and right sides of the component diagram, respectively, and can be specified by an extensible set of *interface* types: Java Interfaces, Web Service Description Language (WSDL), UPnP (Universal Plug & Play), C headers, etc.

SCA integrates different component aspects following the Aspect-Oriented Programming (AOP) paradigm, and provides an eXtensible Markup Language (XML) file format [30] to enable its specification (see Figure 2b). This XML file also represents the overall application architecture, which is defined by the wiring between the references and services of the application components.

Indeed, in addition to the type of interface, the services and references have an associated *binding* type, which indicates the distribution middleware that will be used to connect a service to a reference. An SCA binding encapsulates the complexity of data distribution, and abstracts the application developer from low level communication details. Thus, the developer only specifies the functional interfaces that define the services and references of the application components, and combines these components to design an application. In other words, the developer does not care at all about the actual data distribution technology behind the scenes, which is declaratively defined in the XML file through the *binding* keyword. For instance, on Figure 2b, the service *srvConfig* uses web services while the reference *IDatalogger_Ref* uses Data Distribution Service (DDS).

Currently, the standard includes bindings for SOAP web services, Java Messaging Service (JMS) and Java EE Connection Architecture (JCA), and provides the mechanisms necessary to extend the support to other protocols. In this sense, distributions such as Tuscany [29] or FraSCAti [31] provide bindings for Common Object Request Broker Architecture (CORBA), REST, Remote Method Invocation (RMI) or ATOM. Therefore, an SCA binding is generic, in the sense that it is independent of the service it binds to. It is not necessary to generate interface specific code to invoke a service, unlike in the case of e.g., CORBA or SOAP-WS, where proxy and stub code is generated from an IDL file that defines the service interface. DAMP itself includes a DDS binding which provides extensive QoS support [32] and that has been extended with the security mechanisms introduced in Section 3.3.

The *properties* represent diverse aspects of the component that can be also declaratively configured. They can be seen as component attributes externally accessible. Finally, the component model also supports the specification of constraints and policies in their declarative configuration.

**Figure 3:**
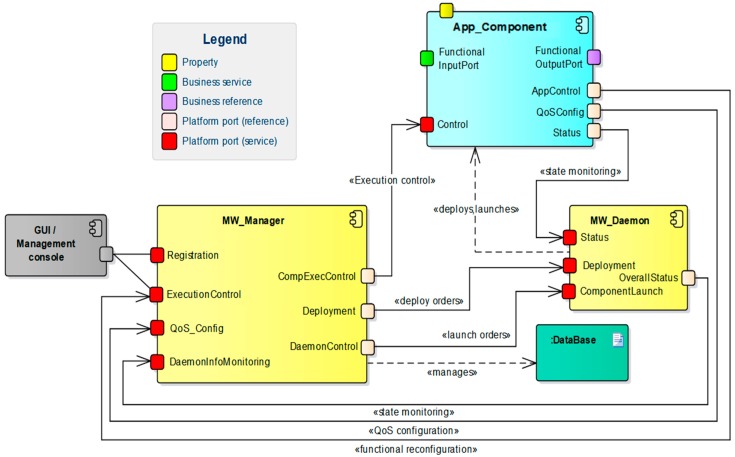
Interconnections among DAMP components.

As commented above, the DAMP platform extends SCA to provide a set of services that fulfill the requirements of the eHealth applications introduced in Section 2. These services are supported upon the architecture described in Figure 3, which is built on three main elements: (1) the *DAMP application component* (*App_Component*), (2) The *node manager (MW_Daemon)* and (3) The *platform manager* (*MW_Manager*). 

**Figure 4:**
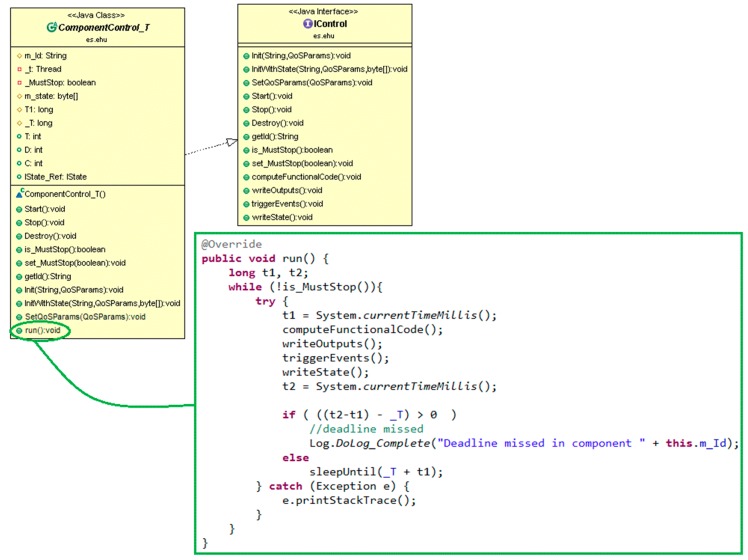
Base class for periodic application components.

A *DAMP application component* is in charge of providing a piece of the application functionality. The application component model extends the SCA component model with one service and two references, which enable their monitoring and control. To achieve such functionality, the application component model provides a base class which implements such service and references. Every application component inherits from this base class, and thus provides the *start*, *stop*, and *initialization* (stateful/stateless) public methods, as depicted in Figure 4. When a periodic component is started, its *run* method is activated, thus initiating the periodic execution of the *computeFunctionalCode*, *writeOutputs*, *triggerEvents* and *writeState* methods. These methods are defined as abstract, as far as they are application specific and must be implemented in the derived component class.

The *platform manager* (*MW_Manager*) provides the high level functionalities such as distributed application monitoring and control, fault tolerance, high availability, or QoS and resource management. The *node manager (MW_Daemon)*, deployed on every infrastructure node, is responsible of controlling the life cycle of the application components that run on its node. It receives the component instantiation orders from the platform manager, and also notifies the manager about the state of the running components.

The system administrator user typically accesses the platform services through a Management console or a Graphical User Interface (GUI). These external elements consume two of the services offered by the MW_Manager: (1) the *registration* service allows application registration and also remote deployment of the application components through the Deployment service provided by the MW_Daemon. (2) The execution control service (*ExecutionControl*) enables the instantiation and execution of the application components, using either the *ComponentLaunch* service provided by the MW_Daemon or the *Control* service implemented by application components.

The rest of the platform services do not require any user action. The application components transfer its internal state to the MW_Manager database through the Status service of their local daemon, thus enabling the overall system monitoring.

**Figure 5:**
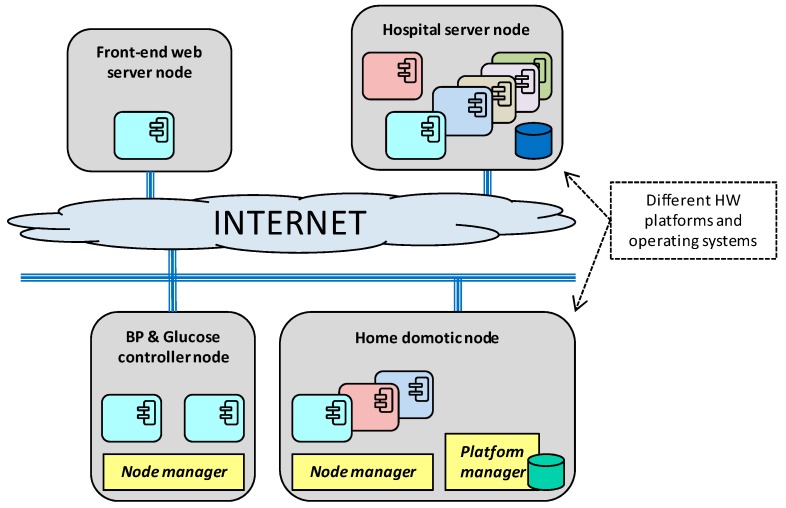
Deployment of the health monitoring application on DAMP.

Figure 5 depicts a specific deployment of the previous example application on the DAMP platform, and reflects some particularities that could converge in a real scenario. In particular, the infrastructure nodes can be shared among several independent applications, and can be diverse in terms of hardware platform and operating system. In this deployment example, the application components that are inside of the home care network are managed by DAMP, whilst the WAN (cloud) components are considered as independent elements maintained by external stakeholders. In this sense, the *hospital database manager server* represents a legacy component that is accessed through a predefined protocol (SOAP-WS), whilst the *web UI* exemplifies a typical web interface susceptible of being outsourced to a cloud service provider.

### 3.2. DAMP Services for Application Management

**Figure 6:**
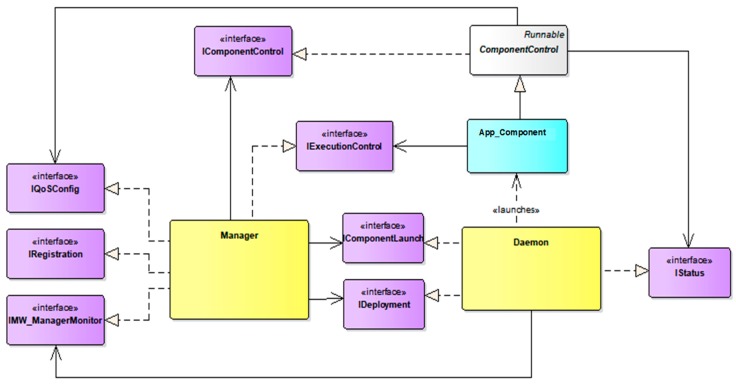
Main elements of the DAMP platform.

The combination of services provided by the aforementioned DAMP elements through a set of defined interfaces enable the fulfillment of the requirements **R1**, **R3**, **R4** and **R6** (collected in Table 1). Figure 6 represents a simplified UML view of the main elements that compose the DAMP platform. The classes that represent the MW_Manager, the MW_Daemon, and the base class ComponentControl are depicted in gray. Every application component (in blue) inherits from this base class. The interfaces implemented by these elements are represented in purple and correspond to the platform services introduced in the architecture.

The *IRegistration* interface represents the functionality offered by the Registration service of the MW_Manager (depicted in Figure 3). It provides the functions to enable the registration of the applications as a set of interconnected components deployed over the distributed nodes that compose the hardware infrastructure of the system (R1 requirement). 

**Figure 7:**
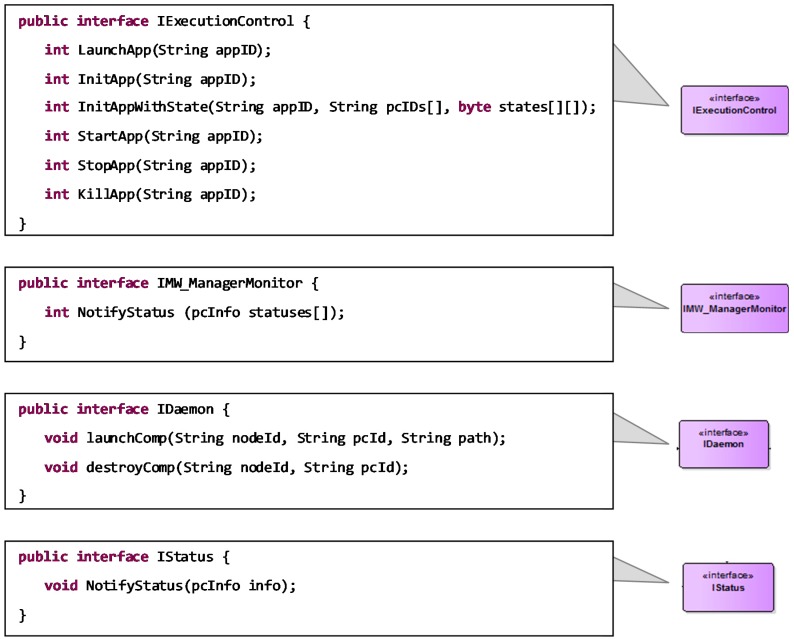
Interfaces for monitoring and control.

The applications installed through the previous interface can be further controlled through the *IExecutionControl* interface, which is depicted in Figure 7. Using this interface, the system administrator can instantiate, initialize, start and stop a registered application. This interface is also accessible to the application components, enabling a functional reconfiguration of applications structure to adapt to context changes, as it will be explained in Section 4. Likewise, the MW_Manager uses the *IDaemon* interface offered by the MW_Daemon to instantiate the application components, as well as to force its termination (R3 and R4 requirements).

Additionally, the *MW_Manager* can be notified about the internal execution state of the application components through the *IMW_ManagerMonitor* interface, which is invoked by the node managers (*MW_Daemon*) that actually perform the application components monitoring. Indeed, the fault tolerance capabilities of DAMP enable stateful application recovery in case of node failure (R6 requirement). This contemplates a non-functional reconfiguration of the system, triggered by a failure event in one of the infrastructure nodes or in an isolated functional component. DAMP achieves the stateful fault tolerance through the following concepts: (1) redundancy of critical application components, (2) continuous monitoring and storage of the components state and (3) autonomous system reconfiguration. 

**Figure 8:**
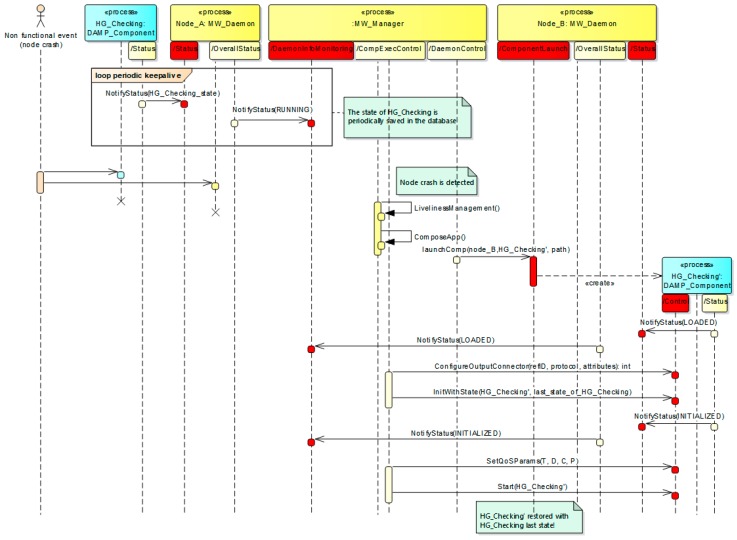
Stateful component recovery use case.

Figure 8 depicts a sequence diagram that describes the stateful recovery of the HG_Checking component in its HG_Checking’ replica, when the node that allocates HG_Checking (Node_A) fails. For simplicity it is assumed that there is only one component on Node_A. Initially, it can be seen how the functional application component communicates its internal state to its local MW_Daemon, after execution. The MW_Daemon packs the states of all the components running in its node and forwards it to the MW_Manager, which finally stores them in the database. The frequency of local state update rate of each component can be different, and it is decoupled from the frequency at which the MW_Daemon notifies the set of states to the MW_Manager. This way the communication between the MW_Daemon and the MW_Manager can be optimized to balance latency vs. throughput, depending on the specific use case. In any case, the MW_Manager uses this periodic reporting as a “keep alive” signal of all the application components running on the node, and thus can detect when a component fails, as far as it stops refreshing its state. In such case of node failure, the MW_Manager checks, for every component, if the component is replicated, and if so, the MW_Manager executes an application composition algorithm to search for a system reconfiguration that includes every replica. This reconfiguration considers the available resources as well as the required QoS levels of the applications that are running in the system [16]. 

If a new feasible system configuration is found, the MW_Manager uses the *IComponentControl* interface provided by the application components to reconfigure the *wiring* of the application components that are antecessors of HG_Checking. This is needed as far as the HG_Checking’ replica is instantiated in another node and thus its service description attributes (e.g., IP or port) differ from the original. Once the bindings (antecessors and also HG_Checking’) are reconfigured, the HG_Checking’ replica is instantiated and initialized with the last state stored in the database, and finally started. It should be noted that the bindings reconfiguration is only needed in case of client-server binding protocols such as e.g., SOAP-WS or REST, where the physical location of the server must be known in order to access it.

### 3.3. Security Considerations

**Figure 9:**
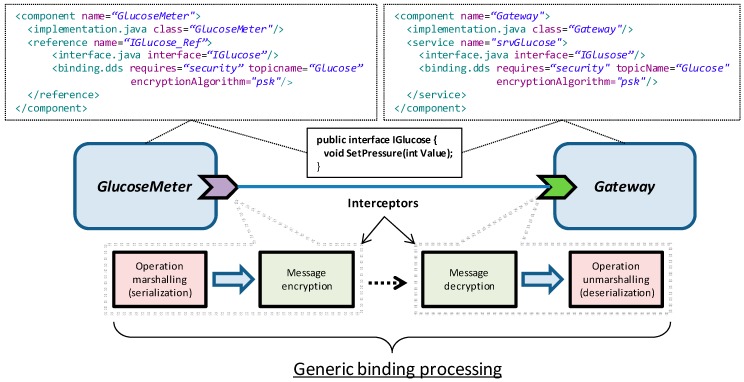
SCA binding processing chain.

To support reliable (**R7**) and secure (**R8**) data transmission between application components, an extension of the DDS binding is proposed. This way, these requirements are both fulfilled by this binding, which can be declaratively configured with safety and/or security needs. Figure 9 shows an example where the binding has been configured as secure, through the *require* keyword. More specifically, the binding has been configured to use a public Pre-Shared Key (PSK) symmetric encryption algorithm.

The implementation of both safety and security aspects relies on the concept of *interceptor* introduced by SCA, which is somehow an AOP approach to solve the transversal requirements that a communication channel can expose. An interceptor is a software module that performs a specific processing (e.g., encryption) over a data stream. Figure 9 represents the processing chain that SCA executes when the GlucoseMeter component invokes a service offered by the Gateway component. This processing chain is represented by the interceptors that in fact compose the binding implementation, and can be seen as a pipeline that sequentially performs several tasks when a service is invoked. In the example there are two output interceptors that perform two tasks in the sender (reference) side: (1) marshal the invocation of the remote procedure call to a serializable format and (2) encrypt this marshaled invocation. In the receiver side (service), two input interceptors perform just the opposite processing, i.e., the decryption and posterior unmarshalling of the invocation message. If both safety and security tags are included in the binding configuration, an additional interceptor must be introduced to integrate the safety mechanisms (CRC, etc.) introduced in [16].

Depending on the selected binding, the serialization format varies. For example, if a SOAP-WS binding (*binding.ws*) is configured for the components interconnection, then the invocation is SOAP formatted (marshaled), whilst in the case of the DDS binding a specific format has been defined to transport the remote procedure call (RPC) invocation. Obviously, this RPC includes the operation name and arguments value and data type. As stated in Section 3.1, the marshalling of an operation invocation is generic, and thus, there is no need of ad-hoc proxy and stub code generation. To support this, SCA provides mechanisms to enable runtime introspection of the functional services [29]. 

The security binding can be configured either with PSK symmetric encryption or with public key infrastructure (PKI) asymmetric encryption. In this latter case an Elliptic Curve Cryptography (ECC) algorithm is used. The asymmetric cryptography provides stronger protection than the symmetric one, but also requires more processing resources. Therefore, asymmetric encryption is usually used to exchange a symmetric key at the handshake stage of communication establishment, which is later used for the subsequent data transmission. Anyway, this is not always the case. For example, in the home LAN side a symmetric encryption could be sufficient to achieve the desired security level, whereas in the public internet this would not be affordable. Depending on the data throughput and link duration, either symmetric or asymmetric encryption could be selected as the best choice.

**Figure 10:**
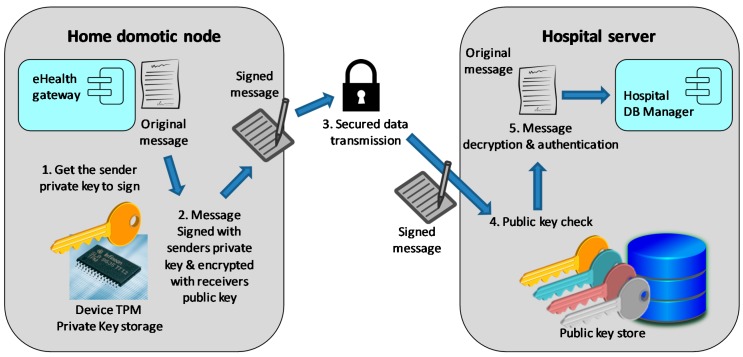
Data encryption and device authentication process.

The proposed security concept relies in the assumption that all the nodes that compose the system infrastructure have a Trusted Platform Module (TPM) installed. A TPM is a dedicated secure crypto processor that integrates the cryptographic keys that are needed for encryption and authentication. Back to the example in Figure 1, let us consider the data transmission process between the eHealth gateway and the hospital database manager components. Figure 10 explains how this process would take place over the proposed DDS security binding, considering the PKI asymmetric encryption option.

When the eHealth gateway component wants to transmit the data corresponding to an hour of compressed physiological data, it transparently uses the secured DDS binding that in turn performs the following steps: first, the private key stored in the node TPM is obtained, and the message is signed with that key. Once signed, the message is encrypted with the public key of the receiver and sent over the network using the DDS middleware. Then, the message is received by the binding interceptor of the hospital DB manager’s service, which first decrypts the message with the hospital private key and then checks the sender public key to authenticate it. If the public key associated with the received message matches any of the public keys stored in the database of trusted devices, the authentication success and the process ends with the delivery of the message to the DB manager component. 

Obviously, every node that allocates application components that are susceptible of receiving input data must have a database with the public keys of the eventual sender components. And vice versa, the senders must be allocated in a node that has registered the public keys of their receivers. This way, the secure communication between them is guaranteed and the requirement **R8** fulfilled. 

### 3.4. Privacy and Availability of Historical Application Data

**Figure 11:**
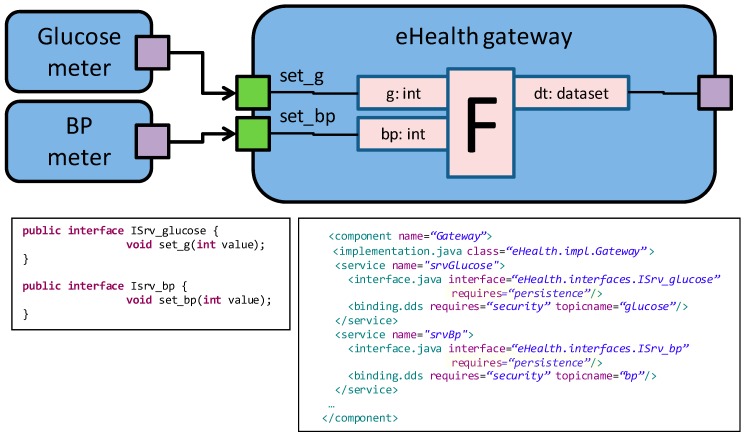
Configurable data persistence.

To support the **R5** requirement, relative to global dissemination of medical data for anonymous big data analysis, the DAMP platform provides a generic mechanism to specify the persistence requirements of input/output data. More specifically, Figure 11 shows how the *persistence* keyword can be associated to a specific interface to declare its persistence needs. When marked as required, the data associated to that interface is automatically persisted by DAMP. 

When a service that requires persistence is invoked, DAMP executes a process that is similar to the stateful fault tolerance mechanism presented in [16]. Every application component features an output port (reference) that is commonly used to transfer its internal state to the platform manager in order to persist it for stateful fault tolerance purposes. In this work, this output port has been extended to support the transfer of internal data in the form of vectors that contain the data name, value, type and timestamp. This form of data representation allows the generic data manipulation and storage mechanisms needed to fulfill the R5 requirement, as far as the time stamped data can be transferred to an external database to perform BDA analysis (the hospital database in the previous example). 

The **R9** requirement is related to the integrity, availability and privacy of this process data. First, the security of the data stored in the platform database is achieved through the application of the same mechanisms that have been discussed in Section 3.2, i.e., data encryption. The platform manager receives the process data from the different application components through the DDS security binding, and remains encrypted in the database. This way, if the database gets compromised, it would be useless without the private key needed to decrypt the data. 

With respect to making data anonymous for BDA, the proposed mechanism is based on the elimination of any relation between the patient and its medical data. This means that any use case which requires maintaining the link between the patient and its personal data (e.g., analysis of personal physiological data) would require additional privacy-protection techniques such as secure multiparty computation or homomorphic encryption, which are out of the scope of this work. Back to the example, when the data is transmitted from the gateway to the hospital, it has no user identification. Indeed, the public key database store of the hospital should not have any relation between the public keys and the patients. This way, the messages that come from a remote device can be authenticated but cannot be certified as belonging to a specific user, thus ensuring data privacy. 

Finally, availability of process data is guaranteed through database replication. As stated in [16], DAMP supports component replication for fault tolerance purposes. These replicas are supported not only for application components, but also for platform specific components, such as the platform manager. Thus, replicating this component, alongside with its database, it is possible to achieve the desired data availability levels.

## 4. ehealth-Centered Design and Development

This section introduces an approach for the design and development of eHealthcare applications, a process where care teams and technology experts must collaborate. On the one hand, physicians know what and when to measure as well as how to interpret these measurements. On the other hand, technology experts include very different profiles, from software developers to network managers or security experts. This separation of concerns is tackled by a domain modeling approach for application specification, which, based on a previous work of authors [17], copes with the main focus of this paper: safety and security requirements of eHealthcare applications. Additionally, this section also proposes a development methodology that fills the gap between the application specification and the running component applications managed by DAMP platform, which is generic in the sense that it does not depend on any application domain. Indeed, as it has been presented, it is suitable for the targeted medical field.

### 4.1. Application Specification

For an application specification it has to be taken into account that, in the end, eHealthcare applications will be composed by a set of software components running under the supervision of a management platform, but it must start with the specification of the *health-centered monitoring (R2)* that defines the customized supervision of a patient, including the detection of the hazardous situations and the corresponding reaction. That is, application specification is leaded by the medical staff although completed by technology experts. With this purpose, the modeling approach consists of a set of interrelated concepts, many of which are initially defined by a physician, and afterwards shared among different technology experts for a detailed characterization. It has to be also considered that each patient suffers from particular diseases such as diabetes, hypertension or cardiopathies, with very different necessities. Even two patients with the same health problem cannot be similarly treated. For example, heart rate thresholds are different for each individual, or the amount of exercise the patient does influenced on the glucose level. 

In order to better understand the proposed modeling approach, let us consider a use case related to the hypoglycemia described in the previous section. If the previous example focused on correlation analysis, the current one completes it as it is aimed at the early prevention of hypoglycemia events in diabetics. In this context, a physician specifies to measure the sweating and heart rate of the patient. In case of abnormal measures during a period of time, apart from warning medical staff, the patient will be asked to measure its glucose level. Finally, if the patient does not respond or the measured level is unsuitable, the physician determines to activate fall detection, which is a right signal of loss of consciousness.

**Figure 12:**
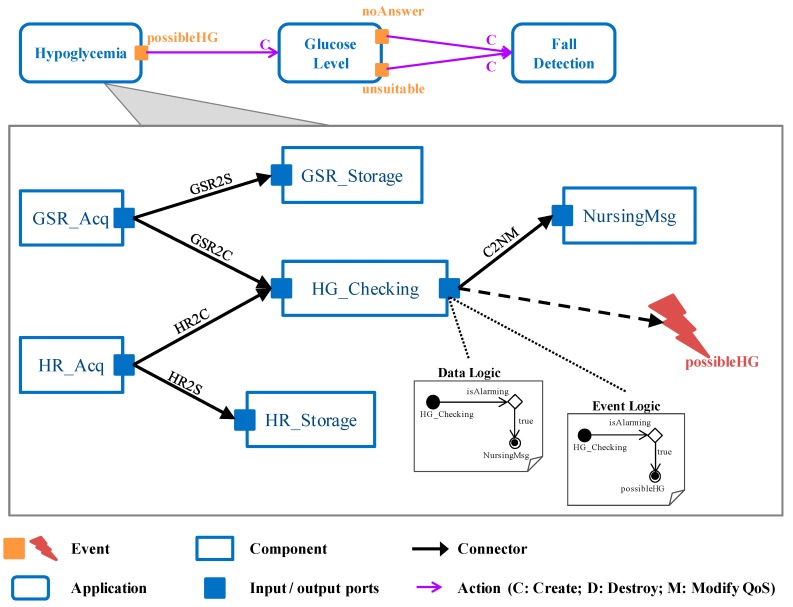
Hypoglycemia prevention scenario.

In the modeling approach every patient is represented by the **Scenario** concept, grouping all the monitoring activities demanded by its health status (**Application** concept). Therefore, in the previous example the scenario for the diabetic patient consists of three applications (see Figure 12): one for identifying a possible hypoglycemia event, another one for checking the current glucose level and the last one for detecting a possible severe case.

Each supervision activity, i.e., application, can be decomposed in several tasks represented by the **Component** concept. For example, the specification of the *Hypoglycemia* application consists of six components (see Figure 12): periodically, the galvanic skin response of the patient (*GSR_Acq* component), as well as its heart rate (*HR_Acq* component) are measured. Both values are stored as part of the patient’s medical history (*GSR_Storage* and *HR_Storage* components, respectively). Additionally, the measures are analyzed together with other previous ones in order to determine if the patient evolution may correspond with a possible hypoglycemic episode (*HG_Checking* component). In that case, apart from warning the medical staff (*NursingMsg* component) the *possibleHG* event is triggered. This involves starting the *GlocoseLevel* application in charge of confirming the alarming situation through the current glucose level of the patient.

It is important to remark that all these components must be customized for the particular patient, as it is presented in Table 2 (properties in blue color). 

Thus, the physician must also determine when the components activate (periodically or after data reception) as well as all the information about the patient needed by the component (e.g., particular thresholds for analyzing the captured data or the patient identifier needed to obtain these data from a local database, which is the case of the example in Table 2).

As components are in charge of different parts of the application functionality, they need to collaborate by exchanging data among them. With this purpose, components are provided with an *Input Port* and/or an *Output Port* for data reception and transmission, respectively. Exchanged data are collected in the *Connector* concept that links the output port of a sending component with the input port of the receiving component. Doctors have to detail both the information required and provided by every component (*Parameter* concept, see Table 2) and how they are connected. In the case of the *GSR_Acq* component, the captured measurement must be sent to the *HG_Checking* (see blue properties in Table 3) and *GSR_Storage* (blue properties Table 4) components. The latter also needs its acquisition time. Note that all the provided parameters are not always sent to all the successor components. Furthermore, data might be sent under certain circumstances that the physician must establish. This *Data Logic* is defined as an activity diagram attached to the output port, based on the parameters provided by the component as a result of its execution. For instance, as it is depicted in the activity diagram of the *HG_Checking* (see Figure 12) component, it communicates with the *NursingMsg* component only when the galvanic skin response and heart rate are too high for the patient. Furthermore, this output parameter (*isAlarming* parameter) is just used for logical decisions as it is not sent to any component.

As previously stated, health-centered monitoring also includes the reaction to abnormal situations, such as a high heart rate and too much sweating, a very low glucose level, or fall detection. The physician makes use of the *Event* concept to identify these relevant situations that demand a reaction and has to define the *Event Logic* that describes the conditions under which the event occurs. Again, the event logic is described by an activity diagram attached to the component in charge of detecting the event, based on its output parameters. As far as reaction is concerned, the modeling approach allows its definition as a set of *Actions* that involve applications. For instance, when the patient status gets worse, i.e., heart rate accelerates and he sweats more, the *possibleHG* event is triggered, which implies starting the *GlucoseLevel* application (see Figure 12, create action). Similarly, when the patient does not respond or the measured level is unsuitable the *noAnswer* and the *unsuitable* events are triggered, respectively, both initiating the *FallDetection* application.

Live data persistence, demanded by the **R5** and **R9** requirements, must also be declaratively defined in the design phase. With this aim, the modeling approach has been extended with properties that allow the care teams to identify which data must be persisted. In particular, the information to be persisted corresponds with the parameters provided by the components, as they constitute their processing results (see Table 3 and Table 4). Additionally, as events represent relevant situations during the supervision of patients, they are also persisted by default. For example, in the first scenario a further analysis of both, the occurrences of the *noAnswer* and *unsuitable* events and the glucose level measured, might allow to study the evolution of the disease in a concrete patient or even infer conclusions at a more global level. Similarly, a deep study of the *possibleHG* events together with normal glucose levels might help in defining a theory for detecting false positives.

Once medical experts have finished applications specification, technology experts complete it by integrating information related to their expertise (green properties in Table 2, Table 3 and Table 4). This is the case of the dependability level of components that must be replicated in order to assure application availability (**R6**). Additionally, it is necessary to indicate if there are stateful components that need to maintain their execution state after a recovery.

Finally, as components collaborate by exchanging data, usually related to sensitive information about patients, technology experts must identify, through properties, if the connectors specified by the medical staff demand safety (**R7**) and/or security (**R8**) support.

### 4.2. Application Development

**Figure 13:**
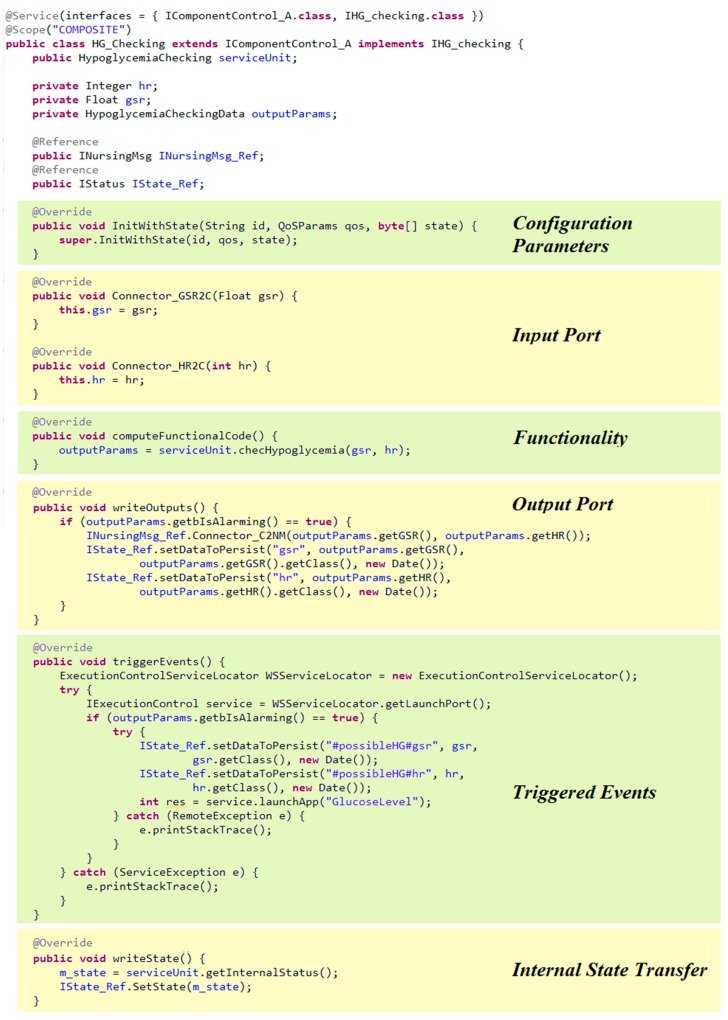
*HG_Checking* component implementation.

The proposed modeling approach allows care teams to play an important role in application specification abstracting them from the particularities of the management platform. Certainly, it is a management platform independent approach. Therefore, it is necessary a methodology for software developers to implement the applications specified by medical experts and that will be managed by the DAMP platform. It is worth noting that the Component concept is the unique modeling concept that becomes source code, therefore it will be the focus of this section. Indeed, the Scenario concept is used by medical experts for structuring applications specification, very useful in large institutions. Therefore, on the basis of the information captured in the application specification, component implementation involves:

- Extending the base class provided by the DAMP platform (see Figure 4) with functional code of the component (see Figure 13).
- Defining the declarative configuration of the component in XML format (as in Figure 11).
- Developing the Java Interfaces needed for data reception (as in Figure 11).

The proposed methodology consists of the following steps, closely related to the modeling concepts described in the previous subsection:

(1) Configuration Parameters

Components must be configured before its first activation. Therefore, the software developer extends the *initWithState* method of the base class to include them (see Figure 4). This method is also used for including all actions needed to initialize the component, e.g., establishing the connection to a database.

(2) Input Port

Data reception is performed through a Java Interface composed by as many methods as Connectors arrive to the port. The arguments of these methods are determined by the connections established during Connector specification. Additionally, in the declarative configuration an input port implies adding an SCA service, taking into account the possibility of persistence demand (see Figure 11).

(3) Functionality

The software developer has to write the code corresponding to the functionality described by the medical experts. It has to be taken into account the required and/or provided parameters previously defined, assigning to them the proper data-type.

(4) Output Port

Data delivery is also performed through Java Interfaces. In this case, the software developer has to add as many references to Java Interfaces as connectors leave the port. Therefore, an output port implies overwriting the *writeOutputs* method of the base class, including the data delivery to all the successor components as well as coding the data logic, if necessary. It is also necessary to add an SCA reference in the declarative configuration.

On the other hand, if the connector has been tagged as persisted, it is necessary to persist all its related output parameters through the reference that is commonly used to transfer its internal state (*IStatus* in Figure 6).

(5) Triggered Events

If the component is in charge of event triggering, the software developer has to overwrite the *triggerEvents* method of the base class. This includes writing the code of the associated logic and invoking the services offered by the DAMP platform for launching, stopping or modifying the QoS parameters of applications. Additionally, as previously stated, triggered events must be persisted by also using the *IState_Ref* reference. In this case, the input parameters that have leaded to the event triggering must be persisted, indicating the associated event.

(6) Internal State Transfer

If it is a stateful component, it is necessary to overwrite the *writeState* method of the base class that manages the component status for availability purposes.

(7) Connectors

Finally, the Connectors of the modeling approach are related to SCA bindings, which are declaratively configured. It is the XML file where the software developer has to indicate if the connector demands safety and/or security (see Figure 11).

As an example, Figure 13 shows the source code of the *HG_Checking* component implementation which has been developed following the previous guidelines, based on the definition of the component and the related connectors (Figure 12, Table 2, Table 3 and Table 4, respectively). As it is depicted in the “Input Port” part of the code, the *gsr* input parameter is received through the *GSR2C* connector, whereas the *hr* input parameter through the *HR2C* connector. Additionally, in the “Triggered Events” part, as a result of the *possibleHG* event detection, the application component invokes the *LaunchApp* method provided by the platform manager (see Figure 7) as a web service. This is the way application components implement the reaction to context changes defined by medical experts. It is important to remark that the *HG_Checking* is a stateful component as it analyzes several sweat and heart rate measures in order to determine a possible hypoglycemia. In this context, a node or component failure may lead to lose data relevant to event triggering, being mandatory to assure its internal state in case of failure recovery. Therefore, before finishing every execution (“Internal State Transfer” in Figure 13) its internal state is transferred to the corresponding node manager, as explained in Section 3.2. Therefore, in case of failure, the recovery process is the one described in Figure 8.

## 5. Assessment

This section presents an assessment of the performance of the DAMP platform, which adds an overhead over the Tuscany SCA reference implementation. This overhead is due to the extra features provided by DAMP, and this section is focused in two of them. On the one hand, DAMP covers the high availability needs of eHealthcare applications (requirement **R6**) through specific fault tolerant mechanisms that enable the stateful system recovery. In this sense, Section 5.1 presents some figures related to application component load times for several bindings and different state sizes. On the other hand, the requirement **R8** related to secure data transmission has been covered by the security binding introduced in Section 3.3. The evaluation of this security binding for different configurations (symmetric vs. asymmetric encryption) is presented in Section 5.2.

### 5.1. Stateful System Recovery

The services to support fault tolerance with stateful system recovery that have been presented in Section 3.2 rely on two mechanisms that have been evaluated from a time consumption perspective. These mechanisms refer to the backup (replica) components load time and their stateful initialization. More specifically, the following aspects have been measured: (1) component load (instantiation) time for different bindings, (2) the influence of a component state size in its initialization time and (3) DAMP overhead vs. pure SCA runtime (Tuscany). It is worth noting that DAMP is currently implemented in Java and thus the comparative analysis has been performed against the Java version of Apache Tuscany SCA. Depending on the machine, the figures may vary, so here the analysis is focused on the comparative analysis between different bindings and state size. 

**Figure 14:**
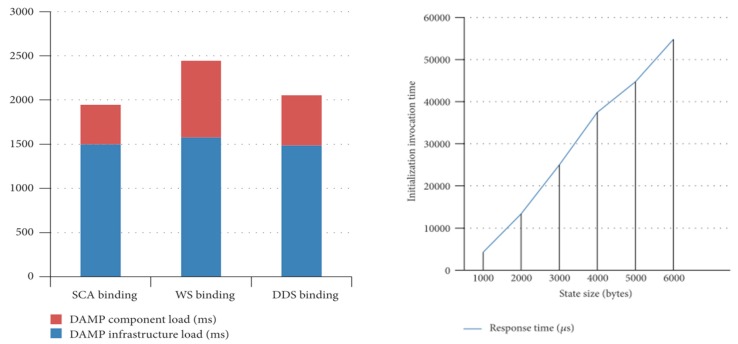
Component instantiation and initialization time.

Figure 14 shows the load time of a component for different types of bindings. The measured times include the load of the Tuscany runtime on which DAMP is based. As it can be seen, the runtime initialization (depicted as *infrastructure load* in the left hand side figure) consumes most of the instantiation time.

It can be also observed that the DDS binding implementation, which is based on RTI´s (Real Time Innovations^®^) distribution, is more efficient than the Tuscany´s native SOAP-WS binding, in terms of component instantiation time. 

**Figure 15:**
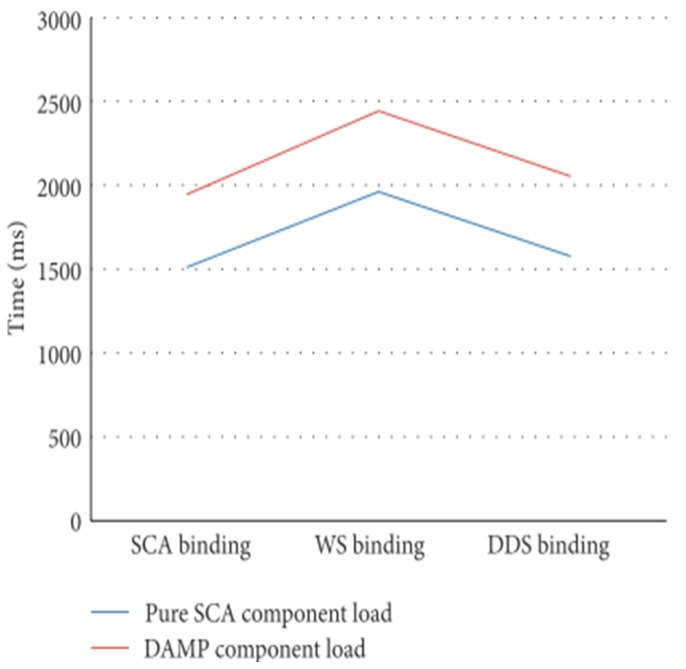
DAMP vs. SCA (Tuscany) overhead.

Figure 15 represents the computing resources overhead that DAMP involves. It has been measured on an application component whose functional services and references have been configured with different binding types. The DAMP application component adds the platform required support ports (*Control*, *QoSConfig* and *Status* ports in Figure 3) to the functional ports. 

Specifically, the graph shows the instantiation time of an SCA component on a Tuscany runtime versus a component with DAMP support. The difference is due to the time involved in the instantiation of the control ports (services and references) that every DAMP application component must provide.

**Figure 16:**
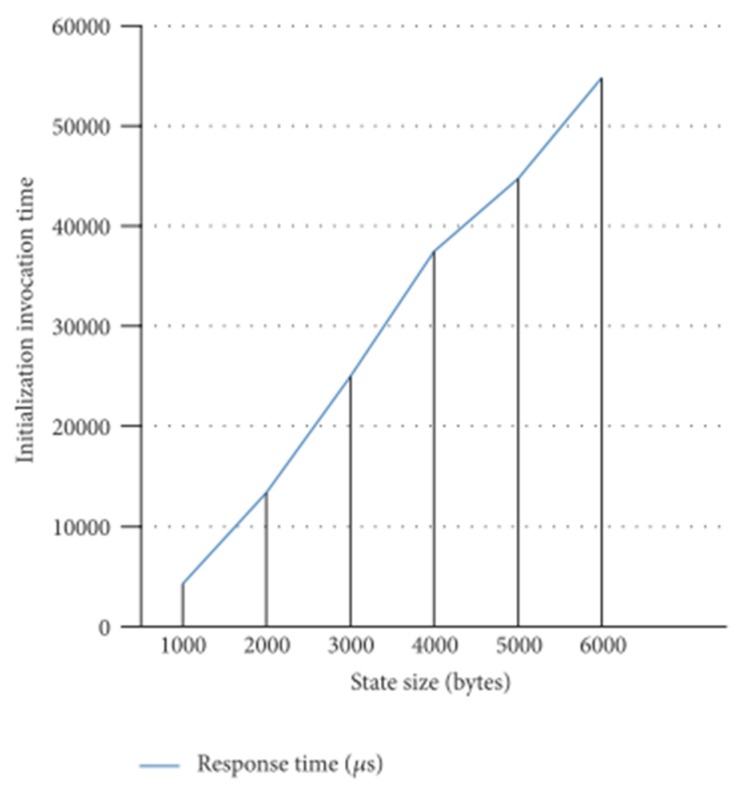
State size influence in component initialization time.

Finally, stateful system recovery implies not only the instantiation of the components that have failed, but also their stateful initialization. The state is restored from the system backup database and injected in the backup components after their initialization. In this sense, Figure 16 reflects the influence of this state size in the component initialization time. It is measured in the MW_Manager, which actually performs the synchronous remote initialization of the application components. The measurements have been performed over a DDS binding and for different state sizes. It can be concluded that the relationship between the size of the state and the initialization time is practically linear.

### 5.2. Security Binding Assesment

**Figure 17:**
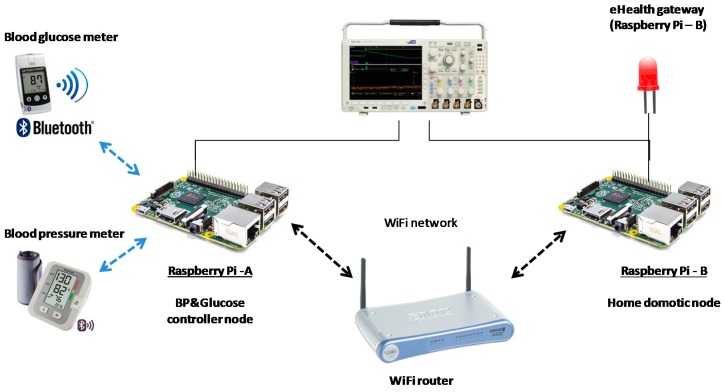
Security performance demonstrator.

To measure the security binding overhead, a demonstrator based on the example application introduced in Figure 1 has been deployed. This demonstrator is shown in Figure 17 and includes two of the nodes depicted in Figure 5: the “Raspberry Pi A” (RPi A) board represents the blood pressure and glucose controller node, whilst the “Raspberry Pi B” (RPi B) board acts as the home domotic node. These nodes allocate the *glucose meter* and the *eHealth gateway* components of Figure 1, which have been used for the performance metrics. The glucose meter component monitors the blood glucose meter connected over Bluetooth to the “Raspberry Pi A”, and uses the secured DDS binding to communicate with the eHealth gateway component over a WiFi network. Finally, the demonstrator includes an oscilloscope to perform the time measurements.

As commented above, the assessment will be focused on characterizing the performance of the DDS binding proposed in Section 3.2, under different security configurations: unsecured, secured with symmetric encryption, and secured with asymmetric encryption. Also, experiments consider both stateless and stateful connections. In a stateless connection neither the sender nor the receiver retains any information related to the connection itself. The message is not acknowledged by the receiver, which receives the packet without any prior connection setup or establishment. Conversely, in a stateful connection, both the sender and the receiver retain information related to the connection state, which can be later used while the connection stays opened [33]. Therefore, in the stateless case, the handshake (i.e., the connection establishment and key interchange) is performed in every message transmission, whilst in the stateful case the handshake is performed once at the beginning of a message sequence transmission, and afterwards several messages can be sent over the same connection. 

The performed tests evaluate the response time (latency) of the security binding, measuring the transmission time span of a secured message between two nodes in a WiFi network. One of the Raspberry Pi boards (A) encrypts a message that is transmitted over the WiFi network to the other Raspberry Pi board (B), which in turn decrypts it. The overhead of the considered security mechanisms is then deduced by comparison between the secured vs. non secured measured transmission times. 

When the Bluetooth glucose meter sends a measure to the BP&Glucose controller node, the glucose meter component running in this node activates one of the Raspberry Pi GPIO pins and forwards the message to the eHealth gateway component running in the home domotic node (Raspberry Pi B), which in turn activates a GPIO pin in Raspberry Pi B, wired to the red led.

The oscilloscope measures the time span between the activation of the GPIO pin in RPi A and the activation of the led attached to the GPIO pin of the receiver board (RPi B). When the RPi A pin is activated, the message containing the measurement is encrypted and sent over the network. When the message is received, it is decrypted and the LED light activated, thus finalizing the measurement. 

**Figure 18:**
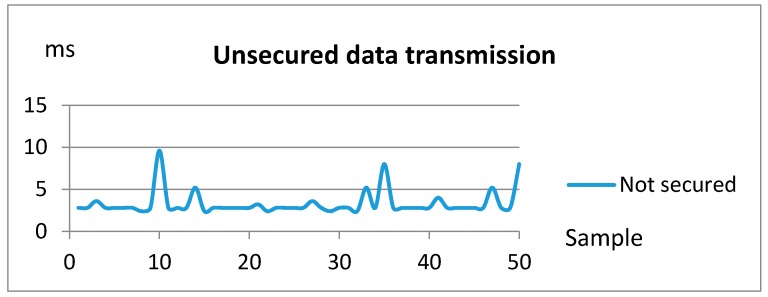
Performance measurements with unsecured data transmission.

Figure 18 represents a set of fifty measurements that have been performed over the previous system without any security encryption. The average of these sample values is 3.31 ms, being the median 2.28 ms and the minimum and maximum values 2.4 and 9.6 ms, respectively. 

Taking these measures into account, the following figures serve as an overhead estimation for the security binding. In this sense, the measures have been performed with different combinations of encryption algorithms and connection types, over the same message size. More specifically, the considered symmetric PSK algorithm has been TLS_PSK_WITH_AES_128_CCM_8, whilst for the asymmetric encryption the TLS_ECDHE_ECDSA_WITH_AES_128_CCM_8 elliptic curve cryptography algorithm has been selected, both registered at the Internet Assigned Numbers Authority [34]. In addition, as previously discussed, both stateless and stateful connection types have been considered. 

It is worth noting that the binding relies on User Datagram Protocol (UDP), which is a lightweight protocol suitable for resource constrained devices, but nevertheless, it is not connection oriented. This is the reason for using Datagram Transport Layer Security (DTLS) to secure the communications. DTLS provides equivalent security guarantees to the SSL/TLS protocol over TCP, enabling both symmetric and asymmetric encryption.

**Figure 19:**
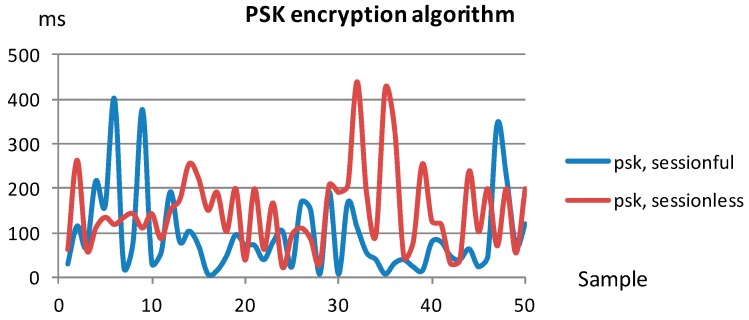
Performance measurements with symmetric data encryption.

Figure 19 displays the measurements corresponding to fifty samples using a symmetric encryption pre-shared key. The sessionful case assumes that the handshake has been performed once at the beginning of the experiment, and thus the handshake related overhead is not included in the figures. This aspect is quite relevant: the average latency in the sessionful experiment is 94.67 ms, whilst the sessionless average latency increases to 150.72 ms, due to the repeating handshake in the fifty message transmissions.

Comparing to the figures related to unsecured data transmission, it can be seen that the transmission latency average values multiplies between thirty and fifty times depending on the session type. This is mainly related to the increment in the computing time that is needed to perform both the encryption and decryption.

**Figure 20:**
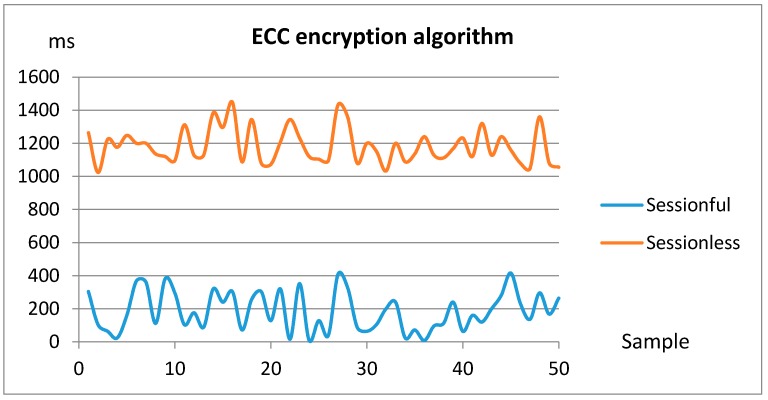
Performance measurements with asymmetric data encryption.

Figure 20 shows the asymmetric data encryption related measures. In this case the latencies grow up considerably, as far as asymmetric encryption consumes much more resources to provide additional features such as authentication and integrity. 

In this case, the differences are greater between the sessionful and sessionless experiments. This is due to the fact that in the sessionful experiment the asymmetric encryption algorithm is only used for initial symmetric private key interchange, which is actually used in the rest of the session. Conversely, in the sessionless experiment every message is encrypted with PKI, which is a highly time consuming task that decreases the performance.

The sessionful test features an average latency of 187 ms, which is not so far from the equivalent symmetric encryption test. Nevertheless, the sessionless asymmetric encryption boost the measured average time in two orders of magnitude with respect to the equivalent symmetric test. As a conclusion, there must be a compromise between security strength and consumed resources. For example, implementing strategies like periodic symmetric key renewal through asymmetric key encryption seems a suitable proposal. It provides an affordable security level whilst saving computation resources and thus improving energy savings in resource constrained devices, which are quite common in eHealthcare applications. In this sense, the proposed approach allows specifying (in the design phase) which components must be secured and which must not, thus limiting the computation resources needed to make the overall application secure. 

## 6. Related Work

This section covers some research work related to the requirements demanded by e-Health applications, mainly focused on their security needs, but also on their life cycle support. From the software engineering point of view, several software architectures have been proposed which consider applications as a set of interacting modules that can be executed on different infrastructure nodes [35,36,37,38,39,40]. In this sense, a component framework defines a component model and a set of tools and methods to ease the management of the applications life cycle, from the initial specification and design, through the implementation, deployment, execution, and final un-installation. 

At runtime, these component frameworks provide runtime engines to support the execution of the application components. These platforms provide mechanisms for, at least, controlling the life cycle of applications, performing their deployment and allowing the communication among application modules (R1, R3 and R4 requirements). Additionally, several platforms have been developed that include the management of some non-functional requirements as those demanded by the target applications [29,41,42], some of which are related to the eHealthcare domain, as in [43,44,45]. 

In this sense, SCA provides extensive support from the interoperability perspective, as far as it includes native support for several communication protocols. Also, it is language agnostic, and thus suitable for the adaptation of legacy code. These characteristics address the requirements related to flexibility, interoperability and heterogeneity. Nevertheless, SCA does not provide in the standard native support for runtime reconfiguration, which is needed to fulfill several requirements such as stateful fault tolerance. FraSCAti, an SCA implementation based on Fractal [46], support dynamic system reconfiguration through scripting, but does not support resource management nor automatic application re-composition in case of non-functional unexpected events such as node failure. This is also the case of other frameworks such as OSGi [47] or SOFA [48]. 

As previously stated health-centered monitoring comprises alarm detection and reaction. In this context, many works do not support adaptation to context changes as they focus on alarm triggering with the aim of warning the patient or asking for medical assistance [49,50,51,52,53]. In this context, as far as authors know, management platforms do not support applications to start the adaptation process as a result of their functional processing. Instead, the reconfiguration process is started by the platform itself [54,55] or upon user demand [56,57,58]. Moreover, they do not allow acting on whole applications, being the changes applied over components On the contrary, the solution proposed in this work goes a step beyond by allowing not only the definition but also the runtime management of relevant events and their corresponding reaction as actions to be executed over applications.

Applications availability is a critical issue in eHealthcare applications as a service failure might result in loss of medical data or application crash. The most used mechanism to avoid service disruption is the redundancy management [59]. Some works provide programming-based solutions [60,61,62], so application definition is subject to application availability. On the contrary, others propose recovery approaches transparent to application definition [43,54,63,64]. In the case of stateful components, many works have focused on determining the best instant to perform the state transfer [65,66,67], which might involve blocking the application execution. In this context, some propose a direct transfer in which the management platform is the responsible of extracting and updating the internal state of the components [67]. Other works propose indirect approaches based on providing components with mechanisms to perform the extraction and updating of their internal state [57,61]. However, the analyzed works do not support state recovery in case of node failure. In this sense, it is necessary an external entity, a management platform, that controls the execution state transfer among components with a global view of the whole system status, as the solution provided in this paper.

On the other hand, the AOP proposed in SCA is an efficient approach for specifying and implementing non-functional properties of application components and their connections, as it separates them from functional goals [68]. The current work takes advantage of this feature in order to assure reliable and secure data transmission and storage.

As far as safety is concerned, reliable communications over a general purpose network such as Ethernet have been considered. In this sense, the IEC 61784-3-3 standard describes mechanisms and measures for a reliable communication over an unsafe transmission media, i.e., a black channel [69]. However, the mechanisms proposed by this standard rely in the application business components, resulting in ad-hoc solutions with low level of abstraction and reusability. In contrast, DAMP platform proposes a generic layer that provides the software developer with safety mechanisms in a transparent way, abstracting it form the communications related issues.

Security is a key research topic when talking about eHealth applications. In this sense, aspects such as confidentiality, integrity or availability must be taken into account [70,71,72,73,74] as they have to be tackled by management platforms at operation time. Several threats have been identified in each of the previous security aspects. For example, confidentiality can be compromised through hacking techniques such as eavesdropping or location and activity tracking. All of them compromise patient´s privacy, providing non authorized access to private medical information [75]. Integrity is another aspect that must be carefully considered, to prevent both non intentional or intentional data modification, which could affect to e.g., medical diagnosis.

To ensure confidentiality, i.e., information hiding to non-authorized users, apart from authentication mechanisms [53,76], several cryptographic techniques have been applied. Depending on the use case, the lighter symmetric cryptography or the heavier asymmetric cryptography can be used [77]. Symmetric cryptography assumes the same secret key for both encrypting and decrypting. Furthermore, the secret key must be shared between the stakeholders by any trustable way. This is why it is known as PSK (pre-shared key). Examples of symmetric encryption algorithms are RC4, DES or AES. Additionally to confidentiality, asymmetric encryption also provides authentication, data integrity and non-revocation. In fact, ensuring that collected data cannot be corrupted is necessary to ensure quality health care [71]. The authentication of the data provider is supported on the concept of data signing. Moreover, the signing process also provides data integrity: if the signed data gets intentionally modified or accidentally corrupted, the sign verification fails. Asymmetric encryption solves the problem of key sharing, as far as each participant owns its own key pair (public/private). Nevertheless, it consumes more computation resources [78]. Examples of asymmetric encryption algorithms are RSA and ECC.

On the other hand, the project presented in [44] focuses on securing communications by concentrating communications to/from the outside through a unique entity that relies on Virtual Private Network (VPN) solutions. Finally, other works like [13] provide a wider overview of the issues that must be taken into account when considering eHealth security. For instance, it points out secure storage of the data and the encryption keys. But it does not provide any specific implementation for the proposed framework, which remains in the conceptual level.

In general, assuring security is a resource consuming task which may be of special interest in the case of working with embedded devices as in the target applications [74,77]. At this point, the current work provides the flexibility necessary to identify and manage those bindings that require security, reducing the system overhead, providing that non-secure networks are protected by other mechanisms such as firewalls [44].

## 7. Conclusions

The Big Data Value Association, a European industry-led organization representing European large and SME industry and research organizations, identifies several technical priorities in its Strategic Research and Innovation Agenda [79]. In particular, it establishes data analytics as a key concept to turn Big Data into value. In this context, it considers data protection and anonymity as a major issue to be addressed. In the draft version 4.0 of the Strategic Research and Innovation Agenda (SRIA), Big Data is considered a key technology to improve the productivity of the healthcare sector and drives its transformation. More specifically, it points out the exploitation of the huge amount of generated medical data as the most effective way to achieve cost savings and at the same time increase the quality of care provided.

In this sense, several technical challenges arise. This research work focuses on two of them: (1) data management, which covers those mechanisms that are necessary to collect and store medical data, as well as make this data available to the medical staff, patients and eHealthcare applications. This medical data will the basis for further eHealthcare improvements based on data exploitation through e.g., data analytics. (2) Data protection covers the privacy, anonymity and authentication needs that sensitive medical data demand. These security related aspects are critical when it comes to enabling medical data mining and at the same time preserving patients privacy, or when end to end security of participating medical devices must be guaranteed.

This research work has identified the requirements of eHealthcare applications, proposing an approach mainly focus on the following ones: health centered monitoring, global diffusion of medical data, and secure data transmission and storage.

Regarding global diffusion of medical data, it has been proved that the application component model enables the specification, in the design phase, of the application data that must be stored for further data analysis and exploitation. At runtime, this data is sent from the application components to the platform manager, which actually stores it in the system database. The confidentiality of this data has been guaranteed through encryption, and its availability needs have been covered by the component redundancy support provided by DAMP. Finally, to preserve the anonymity of the stored medical data when it comes to exploit it in a BDA context, the platform proceeds with the removal of every links between the medical data and the specific patient.

The required security levels in data transmission have been tackled through a DDS binding that incorporates several security features. In particular, the bindings that interconnect the application components services and references can be configured with either symmetric encryption or asymmetric encryption. In the latter case, besides confidentiality, the binding provides authentication and data integrity, as far as the messages are both encrypted and signed.

On the other hand, a modeling approach for application specification has been also presented as a suitable solution for defining health-centered applications. Indeed, the proposed modeling concepts decouple the tasks related to the different stakeholders: medical experts and technology experts. As a result, medical experts focus on specifying remote monitoring applications customized to the particularities of the supervised patients, including also the detection of abnormal situations and the reaction to them. This initial specification is completed by the technology experts who provide information related to availability, safety and security. It is worth noting that security and persistence demands are stated at design time by the corresponding expert, apart from functional considerations. Finally, it has been also proposed a methodology for software developers to implement application components whose execution will be managed by the DAMP platform. This methodology fills the gap between the platform independent application specification and the domain independent management platform.

The binding performance has been evaluated through a demonstrator that implements an eHealth monitoring example application. As expected, the integration of security techniques into the communication involves more resource consumption, which can be considerably high in case of using asymmetric encryption algorithms. To address this problem, the proposed approach allows specifying (in the design phase) which components must be secured and which must not, thus limiting the computation resources needed to make the overall application secure.

Future work will be focused on providing a framework that supports the whole life-cycle of target applications. With this purpose the DAMP platform must be integrated with a model-based tool that allows the graphical specification of the applications, automating the code generation process.

## Figures and Tables

**Table 1:** Requirements of the target applications.

Requirement ID	Requirement Description
R1. Remote monitoring	Remote monitoring of physiological data.
R2. Health-centered monitoring	Health monitoring, alarm detection and reaction customized to the particularities of every patient.
R3. Remote application management	Remote deployment, upgrade and control of eHealth applications.
R4. Heterogeneity and interoperability	The typology of application can be diverse, in terms of implementation language, hardware platforms and operating systems. The interoperability with external systems (e.g., legacy systems) must be supported.
R5. Global diffusion of medical data	Support for the diffusion of digital medical data through a global network infrastructure.
R6. Availability	Availability of critical nodes must be ensured, to achieve adequate dependability levels.
R7. Reliable data transmission	Reliable communication mechanisms over inherently unsafe channels (black channels), including message integrity.
R8. Secure data transmission	Security of the communication channels, including sender identification, authentication and message integrity.
R9. Reliable and secure data persistence	Historical data persistence. The integrity and confidentiality of the database must be guaranteed.

**Table 2:** Characterization of the *HG_Checking* component.

Hypoglycemia Checking (HG_Checking)			
Description	Analyzes if the galvanic skin response of the patient together with its heart rate are related to a possible hypoglycemic episode.		
Activation	After data reception		
Availability Level	1	Is Stateful	Yes
Required Parameters			
Name	hr	Description	Measured heart rate.
Name	gsr	Description	Measure of the galvanic skin response.
Provided Parameters			
Name	isAlarming	Description	Heart rate and galvanic skin response are too high for the patient. Risk of a hypoglycemic episode.
Name	hr	Description	Measured heart rate.
Name	gsr	Description	Measure of the galvanic skin response.
Configuration Parameters			
Name	patientID	Value	31085621
Description	Unique identifier of the patient in the eHealth system			

**Table 3:** Characterization of the *GSR2C* connector.

GSR2C			
Source	GSR_Acq	Target	HG_Checking
Safety	Yes	Security	No
Persisted	No		
Connections			
Output Parameter	galvanicSkinResponse	Input Parameter	gsr

**Table 4:** Characterization of the *GSR2S* connector.

GSR2S			
Source	GSR_Acq	Target	GSR_Storage
Safety	Yes	Security	PSK Symmetric
Persisted	Yes		
Connections			
Output Parameter	galvanicSkinResponse	Input Parameter	gsrValue
Output Parameter	instant	Input Parameter	timeStamp
